# Coriander Oil Reverses Dexamethasone-Induced Insulin Resistance in Rats

**DOI:** 10.3390/antiox11030441

**Published:** 2022-02-23

**Authors:** Mona F. Mahmoud, Noura Ali, Islam Mostafa, Rehab A. Hasan, Mansour Sobeh

**Affiliations:** 1 Department of Pharmacology and Toxicology, Faculty of Pharmacy, Zagazig University, Zagazig 44519, Egypt; dr.nouraali669@yahoo.com; 2 Department of Pharmacognosy, Faculty of Pharmacy, Zagazig University, Zagazig 44519, Egypt; i_m_elbaz@zu.edu.eg; 3 Department of Histology, Faculty of Medicine for Girls, Al Azhar University, Cairo 11751, Egypt; rehababdallah.medg@azhar.edu.eg; 4 AgroBioSciences, Mohammed VI Polytechnic University, Lot 660, Hay MoulayRachid, Ben-Guerir 43150, Morocco

**Keywords:** coriander oil, pancreas, dexamethasone, HOMA-IR, metformin

## Abstract

In the present study, we aimed to investigate the effect of coriander oil on dexamethasone-induced insulin resistance in rats and characterize its chemical composition using gas chromatography-mass spectrometry (GC-MS). Rats were divided into five groups (*n* = 6): Normal control, insulin resistance (IR) control, IR + metformin (50 mg/kg/day, PO, Per Oral), IR + coriander oil low dose (0.5 mL/kg, PO), and IR + coriander oil high dose (1 mL/kg, PO). IR groups were injected with a dose of 10 mg/kg dexamethasone subcutaneously for four consecutive days. All groups received either vehicle or drugs daily for four days. Animal weights and pancreatic weights were measured, and oral glucose tolerance test was performed at the end of study. Fasting glucose, triglycerides (TG), total cholesterol (TC), HDL and insulin levels in serum, MDA, and GSH levels in pancreatic tissue were measured and HOMA-IR was calculated. Immunoexpression of apoptosis markers BAX, and BCL2 was measured in pancreatic tissues and BAX/BCL2 ratio was calculated. Histopathological examination of pancreatic tissues was also performed. Pancreatic weight, serum HDL, pancreatic GSH, and BCL2 were decreased while serum glucose, insulin, TG, TC levels, AUC of OGGT, HOMA-IR, pancreatic MDA, BAX, and BAX/BCL2 ratio were increased in IR rats. Histopathological examination showed congestion, vacuolation and hemorrhage in pancreatic islets. These changes were reversed by metformin and the high dose of coriander oil treatments. The obtained activities could be attributed to the presence of 21 volatile compounds, identified by GC-MS. Our study indicates that coriander oil can be used as an adjuvant antihyperglycemic agent in type 2 diabetes. Further experiments are needed to determine the therapeutic dose and the treatment time.

## 1. Introduction

Diabetes mellitus represents one of the major health problems in the world and is defined as a metabolic disorder that leads to elevation in blood glucose level [1,2]. According to WHO, 422 million persons are diabetic and 1.6 million diabetic cases die each year. Based on diabetes etiology; it can be classified into types 1 and 2 diabetes as major classes. Type 1 diabetes originates from autoimmune disease leading to destruction of pancreatic β-cells and consequently deficiency in insulin while type 2 diabetes attributes to insulin resistance and hence alters the metabolism of carbohydrates, proteins, and lipids [2]. Other causes that can develop diabetes are pregnancy, genetic defects, and drugs, among them glucocorticoids.

The use of dexamethasone, a member of glucocorticoids, is commonly linked to the development of insulin resistance and type 2 diabetes. One of the main reasons of steroid-associated diabetes has been proposed to be pancreatic-cell dysfunction. Because of its selective cell cytotoxicity and capacity to generate hyperinsulinemia and hyperglycemia, dexamethasone is often used in the insulin-resistance diabetic rat model. It can also disrupt cellular metabolic and oxidative processes. Many previous studies investigated the effects of different doses of dexamethasone on pancreas and metabolic changes showed similar results [2,3,4]. Administration of high doses for short duration induce acute hyperglycemia, hyperlipidemia, and insulin resistance. Even low doses of dexamethasone can affect glycemic control by altering glucose transport system and caused steroid diabetes [5]. 

Natural products, among them essential oils, constitute an important source for control and treatment of several illnesses, including diabetes, due to their relatively high safety, bioavailability, and low cost compared to the synthetic analogues [1,6,7]. Coriander oil, derived from *Coriandrum sativum* fruits, has been found to be safe as a food additive and has many biological activities including antibacterial, antifungal and anti-oxidative, anti-inflammatory, hypolipidemic, anxiolytic, and analgesic effects [8,9,10]. Coriander oil could also reduce blood glucose and HbA1c levels, enhance insulin release and protect pancreas against diabetic pathological changes [11]. GC-MS analyses of coriander oils revealed variations in their composition that could be attributed to the variation in their geographical sources and extraction methods; however, linalool was the major component in all of them [9,12,13]. 

In the present work, we characterized the essential oil composition of *C. sativum* fruits using GC-MS as well as investigated its antidiabetic activity in dexamethasone-induced insulin resistance in rats.

## 2. Materials and Methods

### 2.1. Plant Material and Oil Hydrodistillation

*C. sativum* fruits (4 Kg) were purchased from a commercial market at Zagazig city, Sharika, Egypt. They were ground into coarse powder and hydro-distilled using Clevenger type apparatus for 2 h. The oil (12 mL, extraction yield 0.3%) was characterized by an aromatic odor and a pale yellow color and kept in dark vial at the freezer until further analysis. 

### 2.2. GC/MS Analysis 

A Shimadzu GC/MS-QP2010 (Kyoto, Japan) coupled with Rtx^®^-5MS fused bonded column (30 m length, 0.25 mm internal diameter and 0.25 μm film thickness, Restek, Bellefonte, PA, USA) was utilized. The column was initially heated to 45 °C and kept isothermal for 2 min and then the temperature was raised to 300 °C at a rate of 5 °C/min, and kept at 300 °C for 5 min. The injector temperature was 250 °C and helium with flow rate of 1.41 mL/min was used as a carrier gas. The conditions of the mass spectra were 60 mA filament emission current (equipment current), 70 eV ionization voltage, and ion source at 200 °C. The samples were diluted to 1% *v*/*v* and injected using split mode (split ratio 1:15). Retention indices (RI) of the isolated components were calculated with respect to a set of standard *n*-alkanes that were analyzed separately under the same chromatographic conditions.

### 2.3. Experimental Animals

Thirty adult male, Albino Wistar rats, weighing 180–200 g were used. The rats used in the experiment were obtained from Faculty of Veterinary Medicine, Zagazig University, Zagazig, Egypt. Rats were kept under normal light and dark cycle at temperature 21 ± 2 °C. Food and water were given in accordance with the standards (*ad libitum*). 

### 2.4. Induction of Insulin Resistance and Experimental Design

Insulin resistance was induced by a subcutaneous (SC) injection of dexamethasone (10 mg/kg body weight, dissolved in normal saline, injected daily at the same time each day for 4 consecutive days, EPICO Co., 10th of Ramadan City, Egypt) [14]. Rats were randomly divided into five groups (*n* = 6). Normal Control (NC) group: Normal control rats received vehicle, insulin resistance (IR) group: rats received dexamethasone in a dose of 10 mg/kg SC daily for 4 days, IR + metformin group: rats treated with metformin suspended in 10% gum acacia (50 mg/kg/d, PO), IR + coriander oil low dose (CO 0.5) group: rats treated with coriander oil (0.5 mL/kg/d, PO) diluted with olive oil, IR + coriander oil high dose (CO 1) group: rats treated with coriander oil (1 mL/kg/d, PO) diluted with olive oil. The doses of coriander oil and metformin were selected according to previous studies [15]. Vehicle, Metformin, and coriander oil were administrated to rats by oral gavage once daily 1 h before dexamethasone injection for 4 days. Rats were weighed at the beginning and the end of the experiment.

### 2.5. Oral Glucose Tolerance Test (OGTT) and Sample Collection

At day 4, rats were fasted overnight (12 h) for OGTT and the blood glucose level was measured, then 20% glucose was given to the rats, and blood glucose levels were determined again after 30, 60, and 120 min using an automatic blood glucose meter (Bionime, Taiwan) using blood samples from the tail tip. At day 5, rats were fasted again for 8 h and fasting blood glucose level was measured as before. The rats were then anaesthetized by thiopental (40 mg/kg, IP) and blood was collected from orbital sinus, centrifuged for 20 min at 10,000× *g* and serum was stored at −80 °C for biochemical analyses. Thereafter, rats were euthanized by cervical dislocation. The abdomen was opened, and pancreas was dissected and washed with cold saline. A part of pancreas was fixed in 10% neural formalin for immunohistochemical and histopathological analyses, and the other part was snap-frozen in liquid nitrogen and stored at −80 °C for the analysis of tissue oxidative stress markers.

### 2.6. Biochemical Analysis

Serum insulin level was measured by ELISA technique using a rat insulin kit (My BioSource, Co., San Diego, CA, USA, Catalog number, MBS281388). Insulin resistance (IR) index was calculated using homeostasis model assessment (HOMA) index for IR [16] using the following formula: HOMA-IR = blood glucose (mg/dL) × fasting insulin (μU/mL) ÷ 405. Serum triglycerides, total cholesterol, and high-density lipoprotein (HDL) levels were measured colorimetrically using a kit supplied by SPINREACT (Barcelona, Spain). Pancreatic reduced glutathione (GSH) was measured by Rat reduced glutathione ELISA Kit (Catalogue Number: E02G0367) supplied by Shanghai BlueGene Biotech Co., Ltd., Shanghai, China. While malondialdehyde (MDA) in pancreatic tissue was measured by rat malondialdehyde ELISA Kit (Catalog No. LS-F28018) supplied by LifeSpan Biosciences’ (Seattle, WA, USA) according to the manufacturer’s protocol.

### 2.7. Histopathological Study

After suitable fixation in 10% neutral formalin, pancreatic tissue was dried in an alcohol series of 100%, 90%, 70%, 50%, cleaned in xylene, infiltrated, and embedded in paraffin, and then sectioned (5 µm thick) using a rotary microtome for regular histological investigation (LEICA RM 2125, Buffalo Grove, IL 60089, USA). They were deparaffinized and stained with hematoxylin and eosin (H&E) [17]. A light microscope was used to examine the slides (Primo star, ZEISS, Beijing, China). The photographs were shot using a camera (Axiocam ERc 5s, ZEISS, Göttingen, Germany) at Al Azhar University’s Histology Department, Faculty of Medicine for Girls, Al Azhar University, Egypt. Histopathological changes in pancreas were scored, vacuolation, 0–3, congestion, 0–3 and hemorrhage 0–3 and total scores were calculated.

### 2.8. Immunohistochemical Staining

The pancreatic sections were deparaffinized and rehydrated to evaluate the expression of BCL2, an oncoprotein that prevents apoptosis. Then, for 10 min at room temperature, 5% H_2_O_2_ in absolute methanol was added to suppress the activity of endogenous peroxidase. To rinse the sections, phosphate buffered saline (PBS) was used. After that, they were treated with BCL-2 primary antibodies (dilution 1:50; US Biological Life Sciences, US Biological, 4 Technology Way, Salem, Massachusetts 01970, USA). Protein expression was measured using a streptavidin biotin peroxidase kit (Abcam, Cambridge, UK). The tissues were stained with diaminobenzidine (DAB) as a chromogen for BCL2 detection and then counterstained with hematoxylin [18].

To determine the immunoexpression of proapoptotic marker, BAX, 3–4 µm formalin-fixed, paraffin-embedded tissue slices were used for immunohistochemical staining with specific antibodies against BAX (HPA027878, Sigma-Aldrich, Darmstadt, Germany, polyclonal, diluted at 1:100). For visualization, the usual avidin–biotin peroxidase procedure was used with diamino-benzidine (DAB). Mayer’s hematoxylin was used to counterstain [19]. The manufacturer offered a positive slide, and the primary antibody was left out of the negative control sections.

### 2.9. Morphometric Analysis

In ten randomly selected high power microscopic fields, a computerized image system consisting of a Leica Qwin 500 image analyzer coupled to a Leica microscope (Leica, Cambridge, UK) was used to detect the number of BCL2 positive cells and BAX positive cells (in cells of islets of Langerhans). This number was calculated as the number of cells per square meter.

### 2.10. Statistical Analysis

Statistical analyses were accomplished by using the GraphPad Prism 8 software (San Diego, CA, USA). Differences among groups were evaluated by One Way ANOVA test, and the Tukey post hoc test. Two Way ANOVA was used for OGGT test. Spearman’s correlation test was used for correlation analysis. A *p*-value of ˂0.05 was accepted as statistically significant. Histopathological changes were analyzed by One Way ANOVA test, followed by Dunnett’s multiple comparisons test. All data were expressed as means ± standard error of mean.

## 3. Results

### 3.1. Essential Oil Composition

Altogether, 21 volatile compounds, representing 99.63% of the total content of the essential oil, were identified using GC-MS. Linalool dominated the oil and accounted for 75.14% followed by γ-terpinene and α-pinene (5.15% and 4.65%, respectively), Figure S1 and Table S1. Similar pattern was reported before from the Egyptian flora where linalool (accounted for 70.43%), γ-terpinene (accounted for 3.5%), and α-pinene (accounted for 3.96) [9]. 

### 3.2. In Vivo Study Results

#### 3.2.1. Effect of Coriander Oil on Weight and Organ Index of Pancreas 

**Figure 1:**
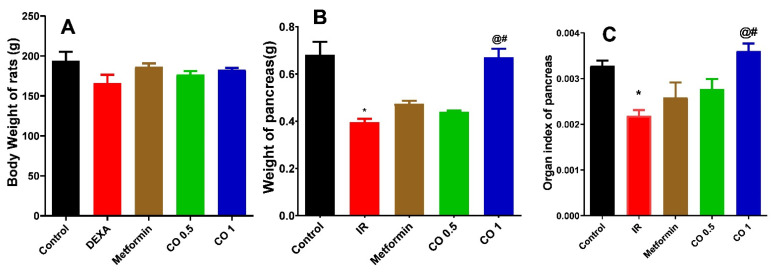
Effect of coriander oil (CO, 0.5 and 1 mL/kg, PO) and metformin (50 mg/kg/day, PO) on body weight (**A**), weight of pancreas (**B**), organ index of pancreas (**C**) in dexamethasone-induced insulin resistance in rats (IR). Results were analyzed by one-way ANOVA followed by the Post-hoc Tukey test. Results are shown in mean ± SEM (*n* = 6). *^, @, #^ *p* < 0.05 compared to normal, IR, and metformin groups, respectively.

There was no significant change in the body weight of animals in dexamethasone group compared to normal control group (*p* > 0.05, Figure 1A). However, dexamethasone significantly decreased the weight and organ index of pancreas in the IR group compared to normal control. Coadministration of coriander oil (The high dose, 1 mL/kg) reversed this action and significantly increased the weight and organ index of pancreas (*p* < 0.05) compared to IR group. Noteworthy, it also significantly increased the weight and organ index of the pancreas (*p* < 0.05) compared to metformin group (Figure 1C).

#### 3.2.2. Effect of Coriander Oil on Oral Glucose Tolerance Test (OGTT)

**Figure 2:**
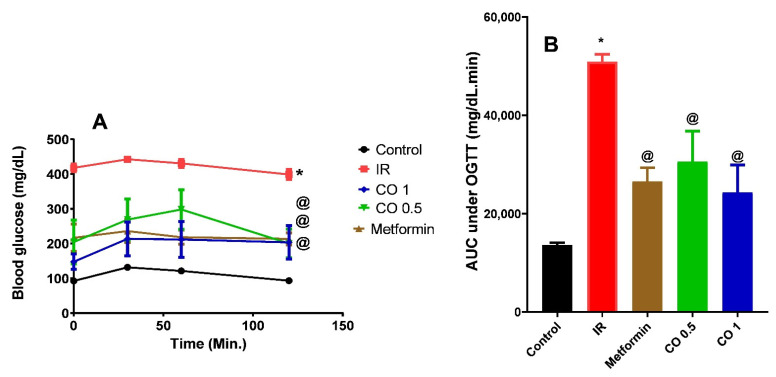
Effect of coriander oil (CO, 0.5 and 1 mL/kg, PO) and metformin (50 mg/kg/day, PO) on oral glucose tolerance (OGTT) test (**A**) and area under the curve (**B**) in dexamethasone-induced insulin resistance in rats (IR). Results were analyzed by one-way ANOVA followed by the post-hoc Tukey test. Results are shown in mean ± SEM (*n* = 6). *^, @^ *p* < 0.05 compared to normal, and IR groups, respectively.

Exogenous oral glucose administration markedly increased the basal glucose level and AUC of glucose in IR group related to normal control (Figure 2, *p* < 0.05). All treatments notably reversed this action and significantly reduced the total AUC of glucose related to IR group (Figure 2). Interestingly, there was no significant difference between metformin and the two dose levels of coriander oil (*p* > 0.05).

#### 3.2.3. Effect of Coriander Oil on Glycemic Parameters

**Figure 3:**
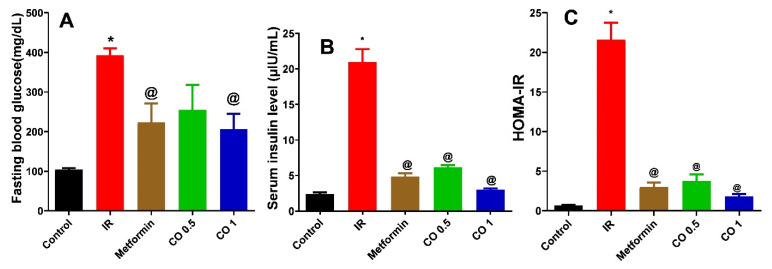
Effect of coriander oil (CO, 0.5 and 1 mL/kg, PO) and metformin (50 mg/kg/day, PO) on fasting blood glucose (**A**), serum insulin (**B**) and HOMA-IR (**C**) in dexamethasone induced insulin resistant rats (IR). Results were analyzed by one-way ANOVA followed by the Post-hoc Tukey test. Results are shown in mean ± SEM (*n* = 6). *^, @^ *p* < 0.05 compared to normal, and IR groups, respectively.

Dexamethasone significantly elevated fasting glucose, serum insulin and HOMA-IR in IR group compared to the normal control (Figure 3, *p* < 0.05). These actions were significantly reversed by metformin, and both dose levels except for fasting glucose where the low dose level (CO 0.5) had no significant effect (*p* > 0.05) compared to IR group (Figure 3A). 

#### 3.2.4. Effect of Coriander Oil on Pancreatic Histology

**Figure 4:**
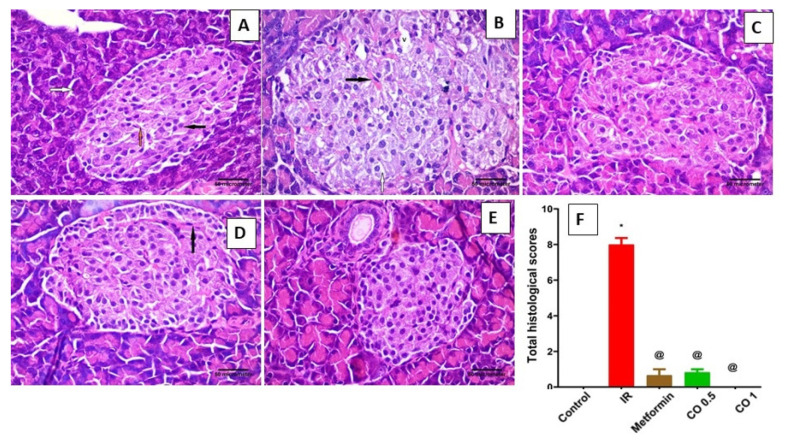
Effect of coriander oil and metformin on pancreatic tissue histology in dexamethasone-induced insulin resistance in rats (IR). (**A**) representative photomicrograph of control rat showing one pale stained islet of Langerhans rich in blood capillaries which are recognized by their flat basophilic endothelial nuclei (orange arrow). Endocrine cells have acidophilic cytoplasm and prominent nuclei at the center (black arrow). The pancreatic acini are lined by irregular triangular cells with basely located rounded nuclei (white arrow). Notice the apical acidophilic granules and the basal basophilia within the acinar cells; (**B**) representative photomicrograph of IR rat showing islet of Langerhans with multiple congested blood capillaries (black arrow), most cells with vacuolated cytoplasm (white arrow). Multiple vacuolations (V) can be seen within the islet; (**C**) representative photomicrograph of metformin (50 mg/kg/day, PO) treated IR rat displaying that the histological structure of the pancreas is like that of the control group; (**D**) representative photomicrograph of low dose coriander oil (0.5 mL/kg, PO) treated IR rat displaying the histological structure of the pancreas is like that in the control group but there is congested blood capillary (white arrow); (**E**) representative photomicrograph of high dose coriander oil (1 mL/kg, PO) treated IR rat displaying that the histological structure of the pancreas is like that in the control group. (H&E, ×400, scale bar = 50 µm). (**F**) column graph represents total histologic scores of pancreases of different groups, data are expressed as mean ± SEM, *n* = 6, *^, @^ *p* < 0.05 compared to normal and the insulin resistance, (IR) groups respectively, by One Way ANOVA followed by Dunnett’s multiple comparisons test.

Table 1 and Figure 4 represent the effects of coriander oil on various histopathological parameters, i.e., congestion, islets morphology, cytoplasmic vacuolation, and hemorrhage. Examination of pancreatic tissues of IR rats showed islet of Langerhans with multiple congested blood capillaries, most cells with vacuolated cytoplasm and multiple vacuolations can be also seen within the islet. In addition, area of hemorrhage can be seen within the islet (Figure 4B). Metformin and coriander oil (the high dose, 1 mL/kg) treatment reversed the above effects on pancreatic islets and showed histological structure of the pancreas like that seen in the control group (Figure 4C,E). The effect of coriander oil on pancreatic tissue histology seemed to be dose dependent where the low dose of coriander oil also showed improvement in pancreatic structure but there were still congested blood capillaries (Figure 4D).

#### 3.2.5. Effect of Coriander Oil on Lipid Profile

**Figure 5:**
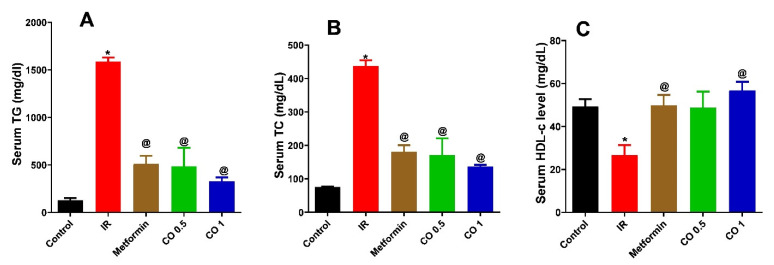
Effect of coriander oil (CO, 0.5 and 1 mL/kg, PO) and metformin (50 mg/kg/day, PO) on serum triglycerides (TG) (**A**) serum total cholesterol (TC) (**B**) and serum high density lipoprotein (HDL) (**C**) in dexamethasone-induced insulin resistance in rats (IR). Results were analyzed by one-way ANOVA followed by the Post-hoc Tukey test. Results are shown in mean ± SEM (*n* = 6). *^, @^ *p* < 0.05 compared to normal, and IR groups, respectively.

Dexamethasone markedly increased TG and TC levels and decreased HDL level in IR group related to control (Figure 5, *p* < 0.05). All treatments notably decreased the elevated levels of TG and TC compared to IR group (Figure 5A, B, *p* < 0.05). However, metformin and coriander oil at the high dose only significantly increased HDL level compared to the IR group (Figure 5C, *p* < 0.05).

#### 3.2.6. Effect of Coriander Oil on Oxidative Stress Markers, MDA and GSH Levels in Pancreatic Tissue Homogenate

**Figure 6:**
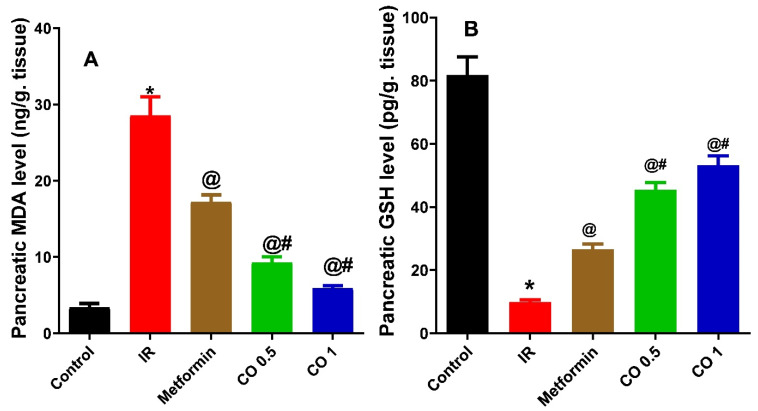
Effect of coriander oil (CO, 0.5 and 1 mL/kg, PO) and metformin (50 mg/kg/day, PO) on pancreatic malondialdehyde (MDA) (A) pancreatic reduced glutathione (GSH) (B) dexamethasone-induced insulin resistance in rats (IR). Results were analyzed by one-way ANOVA followed by the post-hoc Tukey test. Results are shown in mean ± SEM (*n* = 6). *^, @, #^ *p* < 0.05 compared to normal, IR, and metformin groups, respectively.

Dexamethasone markedly elevated the pancreatic lipid peroxidation product, MDA level in the IR group compared to control (Figure 6A, *p* < 0.05). This was noticeably reversed by the reference drug, metformin, and coriander oil treatment (Both dose levels) (Figure 6A, *p* < 0.05). In contrast, dexamethasone significantly declined GSH level in pancreatic tissues homogenate in the IR group compared to control (Figure 6B, *p* < 0.05). This was also evidently reversed with metformin and coriander oil treatment compared to IR group (Figure 6B, *p* < 0.05). Noteworthy, both dose levels of coriander oil exerted more potent effect on MDA and GSH compared with metformin indicating more powerful antioxidant activities (Figure 6, *p* < 0.05).

#### 3.2.7. Effect of Coriander Oil on Pancreatic Apoptosis Markers, BAX, and BCL2 Levels 

**Figure 7:**
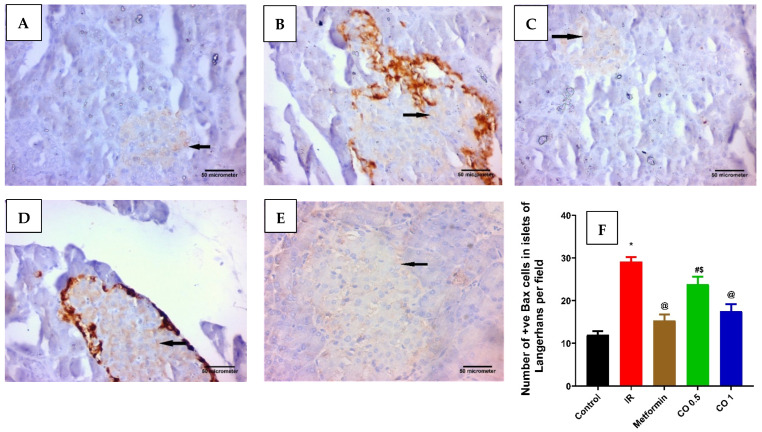
Effect of coriander oil and metformin (50 mg/kg/day, PO) on pancreatic apoptosis marker, BAX in dexamethasone-induced insulin resistance in rats (RI). Photomicrograph of a pancreatic sections of (**A**) the control group displaying few of the islet cells are BAX positive cells; (**B**) the IR group displaying most of the islet cells are BAX positive cells; (**C**) metformin group showing some of the islet cells are BAX positive cells; (**D**) coriander oil (low dose group, 0.5 mL/kg, PO) displaying many of the islet cells are BAX positive cells; (**E**) coriander oil (high dose group, 1 mL/kg, PO) displaying some of the islet cells are BAX positive cells (Avidine biotin peroxidase stain with Hx counter stain ×400, scale bar = 50 µm); (**F**) Bar graph showing the difference in number of immunopositive BAX cells in islets of Langerhans per field in all studied groups which was quantified ×400. Results were analyzed by one-way ANOVA followed by the Post-hoc Tukey test. Results are shown in mean ± SEM (*n* = 6). *^, @, #, $^ *p* < 0.05 compared to normal, IR, metformin, and high dose coriander oil (CO 1) groups, respectively.

**Figure 8:**
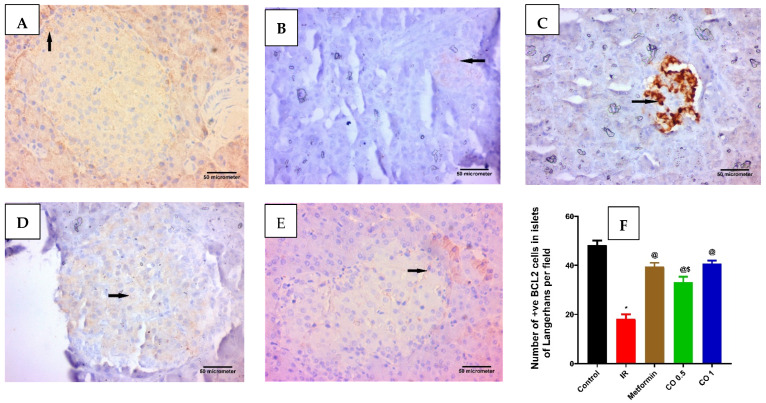
Effect of Effect of coriander oil and metformin on pancreatic anti-apoptotic marker, BCL2 in dexamethasone-induced insulin resistance in rats (RI). Photomicrograph of a pancreatic sections (**A**) control group displaying most of the islet cells are BCL2 positive cells; (**B**) IR group displaying few of the islet cells are BCL2 positive cells; (**C**) metformin group (50 mg/kg/day, PO) displaying many of the islet cells are BCL2 positive cells; (**D**) coriander oil group (low dose, (CO 0.5 mL/kg, PO)) displaying some of the islet cells are BCL2 positive cells; (**E**) coriander oil group (high dose, (CO 1 mL/kg, PO)) displaying many of the islet cells are BCL2 positive cells (Avidine biotin peroxidase stain with Hx counter stain ×400, scale bar = 50 µm); (**F**) Bar graph showing the difference in number of immunopositive BCL2 cells in islets of Langerhans per field in all studied groups that were quantified at ×400. Results were analyzed by one-way ANOVA followed by the post-hoc Tukey test. Results are shown in mean ± SEM (n = 6). *^, @, $^ *p* < 0.05 compared to normal, IR, and high dose coriander oil (CO 1) groups, respectively.

**Figure 9:**
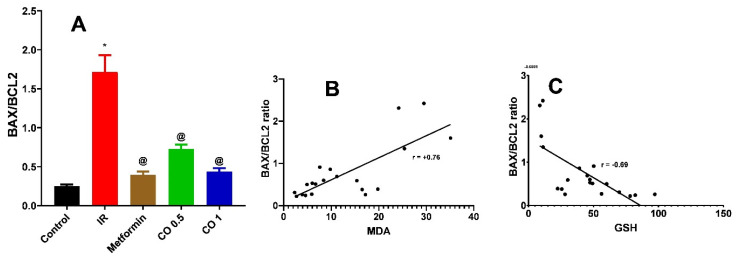
Effect of coriander oil (CO, 0.5 and 1 mL/kg, PO) and metformin (50 mg/kg/day, PO) on BAX/BCL2 ratio (**A**) in dexamethasone-induced insulin resistance in rats (IR). Results were analyzed by one-way ANOVA followed by the Post-hoc Tukey test. Results are shown in mean ± SEM (*n* = 6). *^, @^ *p* < 0.05 compared to normal, and IR groups, respectively. (**B**) Correlation analysis between MDA and BAX/BCL2 ratio. (**C**) Correlation analysis between GSH and BAX/BCL2 ratio.

Dexamethasone considerably increased the pancreatic proapoptotic marker, BAX level in the IR group related to control (Figure 7, *p* < 0.05). This was markedly reversed by metformin and the high dose of coriander oil (Figure 7, *p* < 0.05). In contrast, dexamethasone significantly decreased the antiapoptotic marker, BCL2 level in pancreatic homogenate in the IR group related to control. This was also significantly reversed with metformin and coriander oil (at both dose levels) treatment compared to IR (Figure 8, *p* < 0.05). Noteworthy, coriander oil exerted a dose dependent effect on BAX and BCL2 levels and the high dose of coriander oil had similar effect to metformin on both parameters (Figure 8, *p* < 0.05). Furthermore, BAX/BCL2 ratio was significantly increased in IR group compared to control group, (Figure 9, *p* < 0.05). All treatments significantly decreased this ratio compared to IR group (Figure 9A, *p* < 0.05). Additionally, there was a positive correlation between pancreatic MDA and BAX/BCL2 ratio (r = +0.76 at *p* < 0.0001, Figure 9B). While there was negative correlation between GSH and BAX/BCL2 ratio (r = −0.69 at *p* ˂ 0.001, Figure 9C).

## 4. Discussion

The present study investigates the possible protective role of coriander oil, in comparison with metformin, in dexamethasone induced insulin resistance in rats (type 2 diabetes) and annotates its volatile constituents. Here, compared to normal rats, subcutaneous dexamethasone effectively generated insulin-resistance in normal rats, as demonstrated by hyperglycemia, hyperinsulinemia, impaired oral glucose tolerance, increased HOMA-IR index and hypertriglyceridemia. Previous studies showed that dexamethasone in different dose levels and different durations could induce IR in rats. One of the suggested mechanisms of dexamethasone induced IR is inhibition of hepatic hexokinase activity, inhibition of hepatic glucose oxidation and induction of hepatic gluconeogenesis [20]. Another effect of dexamethasone, that is responsible for development of IR in rats, is decreased expression of GLUT4, the major glucose transporter in adipose tissues and skeletal muscles and subsequent reduction in glucose uptake and utilization that finally leads to the observed hyperglycemia. It was previously reported that dexamethasone increased quantities of free fatty acids in rats that may reduce cell membrane expression of the GLUT-4 transporter, lowering glucose uptake and impairing glucose metabolism in glucose disposal tissues [21,22].

Around the world, *C. sativum* have long been used to treat diabetes and associated inflammation and oxidative stress. Polyphenol fraction of *C. sativum* exerted antihyperglycemic and antihyperlipidemic activities against alloxan induced diabetes in mice [23]. The antidiabetic effect of coriander oil observed in our study may be attributed to the major component of the oil, linalool. It was observed that glucose-metabolizing enzymes, and GLUT-1 expression were all restored after linalool treatment in streptozotocin induced diabetic rats [24]. Linalool also improved glucose uptake by rat diaphragm muscle, inhibited advanced glycation end products formation and improved glucose tolerance in diabetic rats [25].

The administration of high dose dexamethasone also caused hypertriglyceridemia and hypercholesteremia concurrent with reduction of the protective HDL. The observed dyslipidemia in the current study is in accordance with many previous studies [4,25,26,27]. The hyperlipidemia effects of dexamethasone observed in the present study may be attributed to lowering lipoprotein lipase activity and increasing hepatic and intestinal production of very low-density lipoprotein cholesterol (VLDL). Furthermore, dexamethasone promotes hepatic lipogenesis and lipid buildup in the liver and lowers lecithin cholesterol acetyltransferase activity and raises free cholesterol levels. Dexamethasone can also increase circulating fatty acids and enhance lipolysis in adipose tissue [28,29]. As in our study, at high level of dexamethasone, on the other hand, it may be linked to insulin resistance, hyperinsulinemia, an excess of reactive oxygen species (ROS) and hyperglycemia [26,30]. The present study showed that, similar to metformin, coriander oil decreased the elevated triglycerides, serum cholesterol and increased serum HDL levels. However, the low dose of coriander oil failed to elevate HDL level indicating dose dependent effect on HDL levels. Similar to our study, previous works showed that polyphenol fraction of *C. sativum* attenuated hypertriglyceridemia, hypercholesterolemia and decreased HDL levels in alloxan induced diabetes [23]. The improvement in lipid profile by coriander oil, observed in our study, may be related to decreased cholesterol biosynthesis, mostly by inhibition of hydroxy-methylglutaryl -CoA (HMG-CoA) reductase, a major enzyme in cholesterol biosynthesis, and increased cholesterol breakdown to bile acids. Furthermore, oxygen containing monoterpenes, among them linalool, present in coriander oil, may be responsible for its hypolipidemic and hypoglycemic effects. The reduction of triglycerides level by coriander oil may be attributed to decreased TG production and secretion, as well as increased lipoprotein lipase expression and activity.

The previous metabolic derangements caused by dexamethasone were associated with reduction in pancreas weight, increased pancreatic lipid peroxidation and GSH depletion. Previous reports showed that the pancreas size and shape were found to be altered in type 2 diabetes patients. As a result, information on the volume and shape of the pancreas could be useful markers for clinical management and prognosis prediction in diabetic patients. Type 2 diabetes was associated with a small but considerable reduction in pancreas size, as well as an uneven pancreatic border, according to imaging studies [31]. The lack of insulin action on the exocrine pancreas, leading in tissue atrophy, as insulin is a potent growth factor for the exocrine pancreas (disruption of the endocrine and exocrine connection), could explain the reduction in pancreas size in both type 1 and type 2 diabetes patients. Patients with a small pancreas, on the other hand, are more likely to develop diabetes. Moreover, in diabetic patients, immune or inflammation-mediated damage can affect both endocrine and exocrine tissues, resulting in significant parenchymal atrophy. Another possibility, in type 2 diabetes patients, excessive fat infiltration can lead to the replacement of acinar tissues, resulting in pancreas volume loss [32]. In the current study the observed reduction of pancreatic weight may be attributed to the lack of insulin action on exocrine pancreas because of developed insulin resistance observed in our study and/or excessive fat infiltration replacing acinar tissues as dexamethasone administration was associated with significant hyperlipidemia. Noteworthy, only the high dose coriander oil restored pancreatic weight to normal value. This effect required further investigation. 

The increased oxidative stress in dexamethasone treated pancreas is in accordance with previous studies [33]. Dexamethasone also could increase oxidative stress in other tissues like skeletal muscle via glucocorticoid receptor [34]. Another mechanism of elevated lipid peroxidation and depleted glutathione in pancreatic tissues by dexamethasone is hyperglycemia and glucotoxicity of pancreatic islets observed in the current study. Coriander oil at both dose levels was better than metformin in reducing lipid peroxidation and restoring depleted glutathione in pancreatic tissues. The antioxidants effects of coriander oil may be attributed to its high linalool content. Previous studies showed that linalool reverses benzene-induced cytotoxicity, oxidative stress, and lysosomal/mitochondrial damage in human lymphocytes [35]. Furthermore, linalool can protect rats hippocampus from oxidative stress and gliosis caused by Aβ1-42 in rat model of Alzheimer’s disease via increasing expression of Nrf2 and HO-1 [36]. 

Dexamethasone, in the present study, induced oxidative stress led to apoptosis of pancreatic islets. Moreover, the apoptotic markers such as BAX, BAX/BCL2 ratio were increased and were positively correlated with lipid peroxidation product, MDA and negatively correlated with GSH. Furthermore, the antiapoptotic marker, BCL2 was substantially decreased in dexamethasone treated rats. The present study is in accordance with Suksri et al. [37] who showed that dexamethasone induced apoptosis of pancreatic β-cells tumor necrosis factor-related apoptosis-inducing ligand (TRAIL) pathway. Furthermore, they found that superoxide generation, caspase-8, -9, and -3 activity, NF-B, and Bax were all elevated by dexamethasone, whereas BCL2, an anti-apoptotic protein, was suppressed which confirms our study findings. Another mechanism of dexamethasone induced apoptosis is endoplasmic reticulum stress-mediated apoptosis at high doses of dexamethasone [38]. It was observed that the essential oils of lavender and coriander, as well as linalool, reduced the formation of intracellular reactive oxygen species and the activation of the pro-apoptotic enzyme caspase-3, in neuronally differentiated PC12 cells exposed to Aβ1-42 oligomers [39]. In accordance with the earlier study, we showed that coriander oil, dose dependently, suppressed apoptosis and increased anti-apoptotic protein BCL2. The high dose of coriander oil exerted similar effects to metformin. The antiapoptotic potential of coriander oil may be attributed to its antioxidant and anti-inflammatory effects [9]. 

## 5. Conclusions

This study is first to our knowledge that shows dose dependent antidiabetic effects of coriander oil in comparison with metformin and its underlying mechanism of action. The present study reported that the high dose of dexamethasone may lead to insulin resistance, dyslipidemia, and pancreatic islets apoptosis via increasing oxidative stress. Coriander oil can reverse the effects of dexamethasone comparable to metformin. Therefore, it is suggested that coriander oil can be used as an adjunctive antihyperglycemic agent in diabetes, especially steroid induced diabetes. However, uncovering more about the underlying mechanisms requires further study.

## Figures and Tables

**Table 1:** Histopathological changes in pancreas of different groups.

Groups	Vacuolation	Congestion	Hemorrhage	Total Scores
Control	0.00 ± 0.00	0.00 ± 0.00	0.00 ± 0.00	0.00 ± 0.00
IR	2.83 * ± 0.17	2.50 * ± 0.22	2.67 * ± 0.21	8.00 * ± 0.37
Metformin	0.33 ^@^ ± 0.21	0.33 ^@^ ± 0.21	0.00 ^@^ ± 0.00	0.67 ^@^ ± 0.33
Coriander oil (0.5 mL/kg)	0.17 ^@^ ± 0.17	0.67 ^@^ ± 0.21	0.00 ^@^ ± 0.00	0.83 ^@^ ± 0.17
Coriander oil (1 mL/kg)	0.00 ^@^ ± 0.00	0.00 ^@^ ± 0.00	0.00 ^@^ ± 0.00	0.00 ^@^ ± 0.00

Data were expressed as the mean ± standard error, *n* = 6. ∗ *p* < 0.05 compared to the control group; ^@^ *p* < 0.05 compared to the insulin resistance, (IR) group by One Way ANOVA followed by Dunnett’s multiple comparisons test.

## Data Availability

All data are presented at the manuscript.

## Supplementary Materials

The following supporting information can be downloaded at: https://www.mdpi.com/article/10.3390/antiox11030441/s1, Figure S1: GC-MS profile of *C. sativum* fruits oil. Table S1: Essential oil composition of *C. sativum* fruits using GC-MS analysis.
