# Correction: Mahmoud et al. Coriander Oil Reverses Dexamethasone-Induced Insulin Resistance in Rats. *Antioxidants* 2022, *11*, 441

**DOI:** 10.3390/antiox13111410

**Published:** 2024-11-18

**Authors:** Mona F. Mahmoud, Noura Ali, Islam Mostafa, Rehab A. Hasan, Mansour Sobeh

**Affiliations:** 1 Department of Pharmacology and Toxicology, Faculty of Pharmacy, Zagazig University, Zagazig 44519, Egypt; dr.nouraali669@yahoo.com; 2 Department of Pharmacognosy, Faculty of Pharmacy, Zagazig University, Zagazig 44519, Egypt; i_m_elbaz@zu.edu.eg; 3 Department of Histology, Faculty of Medicine for Girls, Al Azhar University, Cairo 11751, Egypt; rehababdallah.medg@azhar.edu.eg; 4 AgroBioSciences, Mohammed VI Polytechnic University, Lot 660, Hay MoulayRachid, Ben-Guerir 43150, Morocco

In the original publication [1], there was a mistake in Figures 7 and 8 as published. Figure 7A was mistakenly used again for Figure 7E; Figure 7D was mistakenly used again for Figure 8A; and Figure 7B was mistakenly used again for Figure 8E. The corrected Figure 7 and Figure 8 appear below. 

The authors state that the scientific conclusions are unaffected. This correction was approved by the Academic Editor. The original publication has also been updated.

## Figures and Tables

**Figure 7:**
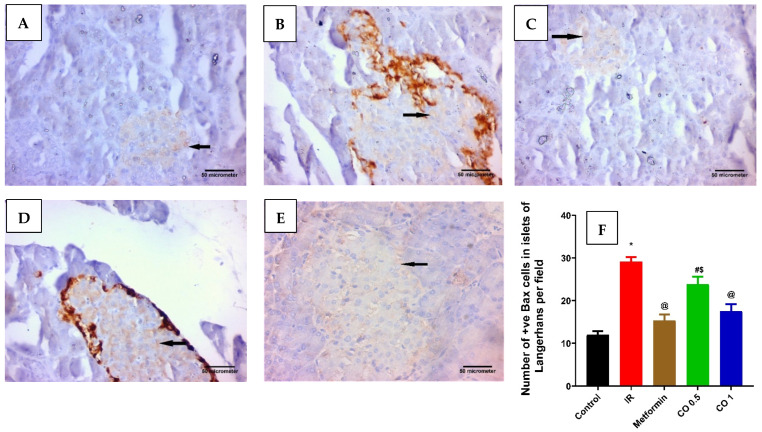
Effect of coriander oil and metformin (50 mg/kg/day, PO) on pancreatic apoptosis marker, BAX in dexamethasone-induced insulin resistance in rats (RI). Photomicrograph of a pancreatic sections of (**A**) the control group displaying few of the islet cells are BAX positive cells; (**B**) the IR group displaying most of the islet cells are BAX positive cells; (**C**) metformin group showing some of the islet cells are BAX positive cells; (**D**) coriander oil (low dose group, 0.5 mL/kg, PO) displaying many of the islet cells are BAX positive cells; (**E**) coriander oil (high dose group, 1 mL/kg, PO) displaying some of the islet cells are BAX positive cells (Avidine biotin peroxidase stain with Hx counter stain ×400, scale bar = 50 µm); (**F**) Bar graph showing the difference in number of immunopositive BAX cells in islets of Langerhans per field in all studied groups which was quantified ×400. Results were analyzed by one-way ANOVA followed by the Post-hoc Tukey test. Results are shown in mean ± SEM (*n* = 6). *^, @, #, $^ *p* < 0.05 compared to normal, IR, metformin, and high dose coriander oil (CO 1) groups, respectively.

**Figure 8:**
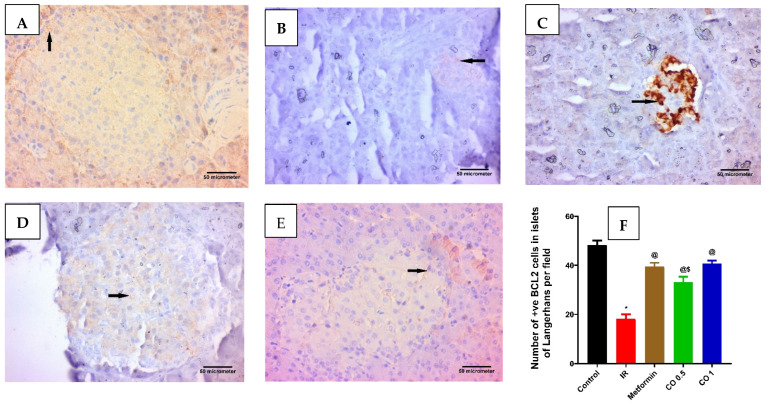
Effect of Effect of coriander oil and metformin on pancreatic anti-apoptotic marker, BCL2 in dexamethasone-induced insulin resistance in rats (RI). Photomicrograph of a pancreatic sections (**A**) control group displaying most of the islet cells are BCL2 positive cells; (**B**) IR group displaying few of the islet cells are BCL2 positive cells; (**C**) metformin group (50 mg/kg/day, PO) displaying many of the islet cells are BCL2 positive cells; (**D**) coriander oil group (low dose, (CO 0.5 mL/kg, PO)) displaying some of the islet cells are BCL2 positive cells; (**E**) coriander oil group (high dose, (CO 1 mL/kg, PO)) displaying many of the islet cells are BCL2 positive cells (Avidine biotin peroxidase stain with Hx counter stain ×400, scale bar = 50 µm); (**F**) Bar graph showing the difference in number of immunopositive BCL2 cells in islets of Langerhans per field in all studied groups that were quantified at ×400. Results were analyzed by one-way ANOVA followed by the post-hoc Tukey test. Results are shown in mean ± SEM (n = 6). *^, @, $^ *p* < 0.05 compared to normal, IR, and high dose coriander oil (CO 1) groups, respectively.
