# Supplementary material for: Coriander Oil Reverses Dexamethasone-Induced Insulin Resistance in Rats
Source: Antioxidants (Basel). 2022 Feb 23;11(3):441. doi: 10.3390/antiox11030441 (PMC8944706; doi:10.3390/antiox11030441)
Supplement: Supplementary file 1 [file antioxidants-11-00441-s001.zip › antioxidants-1548331-supplementary.pdf]

Coriander oil reverses dexamethasone-induced insulin resistance in rats

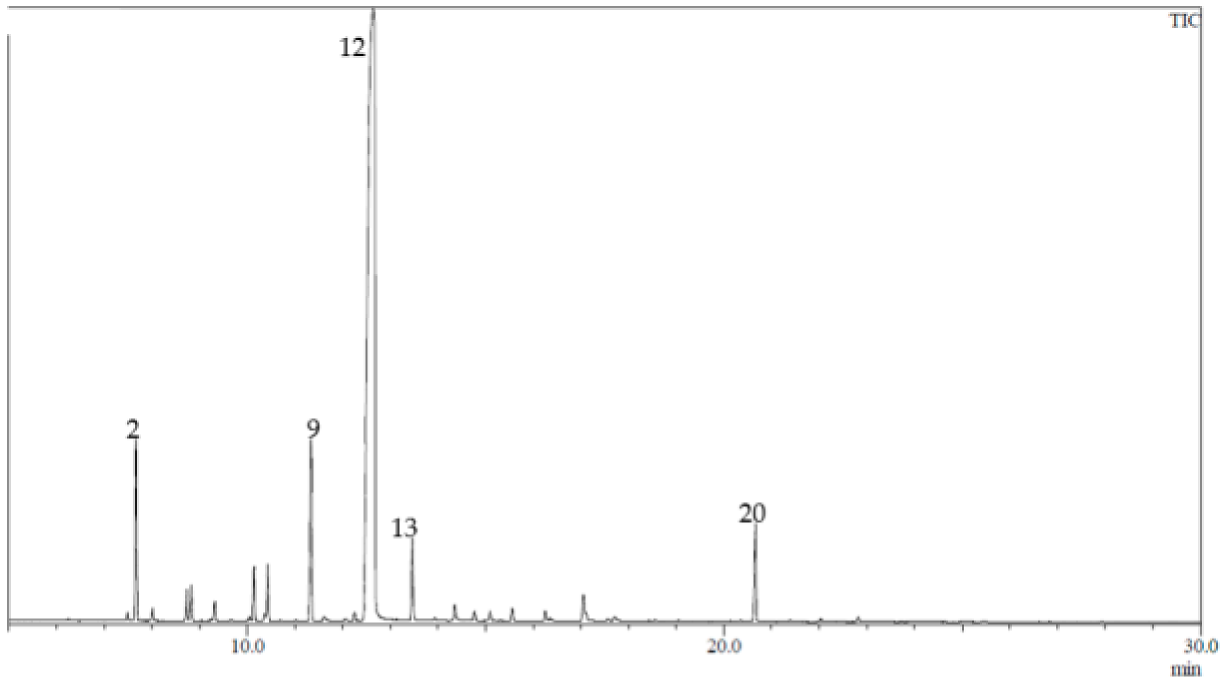

Figure S1. GC-MS profile of *C. sativum* fruits oil.

Table S1. Essential oil composition of *C. sativum* fruits using GC-MS analysis.

No.	Compounds	Calculated retention index	Relative abundance (%)
1	Thujene	921	0.21
2	α-Pinene	928	4.65
3	Camphene	941	0.31
4	Sabinene	967	0.84
5	β-Pinene	970	0.97
6	Myrcene	988	0.47
7	*p*-Cymene	1016	1.49
8	Sylvestrene	1025	1.67
9	γ- Terpinene	1054	5.15
10	cis linalool oxide	1063	0.2
11	Terpinolene	1084	0.25
12	Linalool	1096	75.14
13	Camphor	1123	2.4
14	Isoborneol	1151	0.59
15	Terpinen-4-ol	1164	0.29
16	α-Terpineol	1175	0.29
17	Decanal	1190	0.35
18	Cumin aldehyde	1213	0.27
19	Geraniol	1241	1.08
20	Geranyl acetate	1368	2.91
21	Caryophyllene E	1418	0.1
Total identified			99.63
Monoterpene hydrocarbons			16.01
Oxygen containing monoterpenes			82.9
Others			0.72
